# Anti-Arthritis Effect of Ethanol Extract of Sacha Inchi (*Plukenetia volubilis* L.) Leaves Against Complete Freund’s Adjuvant-Induced Arthritis Model in Mice

**DOI:** 10.21315/tlsr2023.34.3.13

**Published:** 2023-09-30

**Authors:** Thi Phuong Nhung Tran, Thi-Trang Nguyen, Gia-Buu Tran

**Affiliations:** 1 Department of Biotechnology, Institute of Biotechnology and Food Technology, Industrial University of Ho Chi Minh City, 12 Nguyen Van Bao Street, Go Vap District, Ho Chi Minh City, Vietnam; 2 Department of Food Science and Nutrition, Institute of Biotechnology and Food Technology, Industrial University of Ho Chi Minh City, 12 Nguyen Van Bao Street, Go Vap District, Ho Chi Minh City, Vietnam; 3 Faculty of Pharmacy, Ton Duc Thang University, 19, Nguyễn Hữu Thọ, Tân Hưng, Quận 7, Thành phố, Ho Chi Minh City, Vietnam

**Keywords:** Anti-Arthritis Effect, Antioxidants, Inflammatory Cytokines, Animal Model, *Plukenetia volubilis*

## Abstract

Sacha inchi (*Plukenetia volubilis* L.) is a well-known oleaginous plant used as food source and traditional medicine by indigenous people for a long time. This study was conducted to evaluate anti-arthritis effect of ethanol extract of Sacha inchi leaves and provide scientific evidence to develop the new anti-arthritis remedy from Sacha inchi. Rheumatoid arthritis model was established by injection of complete Freund’s adjuvant into right hind footpads of mice and three doses of ethanol extract of Sacha inchi leaves (100, 200 and 300 mg/kg body weight) were used for treatment. The severity of arthritis was evaluated by measuring the ankle diameter and arthritic score, hematological and biochemical parameters (erythrocytes, leukocytes, lymphocytes, monocytes, granulocytes counts, erythrocyte sedimentation rate, C-reactive protein and rheumatoid factor). The pro-and anti-inflammatory cytokines (TNF-α, INF-γ, IL-1β, IL-6, and IL-10) and the histology change of joint were also examined. All three doses of extracts significantly alleviated ankle diameter and arthritic score. Furthermore, the extracts could ameliorate the alternation of inflammatory cytokines as well histological features of CFA-induced mice. The efficacy of extract dose of 300 mg/kg body weight is comparable with reference drug (Mobic, 0.2 mg/kg body weight). This study indicates Sacha inchi leaf extract as the promising remedy for treatment of arthritis.

**Highlights**

- In Vietnam folk medicine, *Plukenetia volubilis* is used as an anti-arthritis remedy but the scientific evidence and mechanism are still lacking.
- *P. volubilis* leaves extract exhibites a remarkable anti-arthritis effect on mice and modulates production of inflammatory cytokines.
- *P. volubilis* is a potential therapeutic medicine for treatment of rheumatoid arthritis.

## INTRODUCTION

Rheumatoid arthritis (RA) is an autoimmune disorder characterised by chronic inflammation in several minor joints of wrists, hands, fingers and feet, resulting in immune cells infiltration, erosion of cartilage and bone, as well as synovial hyperplasia in the patient’s synovium (Han *et al*. 2022; Guo *et al*. 2018). It not only causes pain, swelling, and functional disability at joints but also results in some systematic consequences such as cardiovascular disorders, fibrotic diseases and hematologic and kidney cancer (Guo *et al*. 2018). Patients with RA are also suffering from a higher risk of disability and mortality, as well as a higher healthcare expenditure, especially over the first 10 years of the initiation of the disease (Choi *et al*. 2019). Additionally, Safiri *et al*. (2019) proposed RA as one of the major global public health burdens with the estimation of the age–standardised point prevalence and annual incidence rates of about 246.6 and 14.9 in 2017, which have a tendency to increase during recent years. A variety of therapies have been developed for RA treatment, from conventional disease-modifying anti-rheumatic drugs (DMARDs), such as methotrexate, leflunomide, sulfasalazine, hydroxychloroquine, as well as biological DMARDs targeting specific molecules or molecular pathway associated with RA inflammatory response (Infliximab, Tocilizumab, Abatacept, Rituximab, Anakinra, etc.), to novel potential small molecules including Jakinibs, Baricitinib, Tofacitinib (Han *et al*. 2022; Guo *et al*. 2018). To date, the American College of Rheumatology and the European League Against Rheumatism propose the regime of methotrexate (25 mg/week) plus glucocorticoids as the first-line medicines to treat RA (Han *et al*. 2022). However, there is a considerable portion of RA patients do not respond well to the available treatments, and many adverse effects during long-term exposure to DMARDs have observed (Guo *et al*. 2018; Turiel *et al*. 2010). Therefore, the discovery of alternative approaches to conventional and modern medicines that not only have higher effectiveness but also fewer adverse effects is a crucial task, especially from traditional medicinal plants which have been well-known resources for a long time about their effectiveness and safety (Galehdar *et al*. 2018). Recently, some research has highlighted the application of herbal medicines or formulations, including Sesamum indicum, Plantago major, and Ayurvedic formulation Vatari Guggulu as supplements for the treatment of RA (Ruckmani *et al*. 2017; Triastuti *et al*. 2021; Patel & Pundarikakshudu 2015).

Sacha inchi (*Plukenetia volubilis* L.) is an oleaginous plant of the Europhobiaceae family which is originated from the Amazon basin and then cultivated in several Asian countries such as China, Thailand, Vietnam, Cambodia, Laos, etc., as well as South and Central America as an edible crop (Cárdenas *et al*. 2021; Kodahl & Sørensen 2021). Sacha inchi seed oil also has been used as a traditional remedy in Peruvian ethnomedicine, especially for the treatment of cutaneous wounds, aching muscles, and rheumatoid arthritis (Cárdenas *et al*. 2021; Kodahl & Sørensen 2021; Chirinos *et al*. 2013). Recently, some research suggested that Sacha inchi possesses some bioactivities and beneficial effects such as antioxidant, antimicrobial, and anti-cancer effects, as well as alleviating hypertension (Cárdenas *et al*. 2021; Nascimento *et al*. 2013; Li *et al*. 2020; Gonzalez-Aspajo *et al*. 2015). Recently, Tran and Tran (2021) have proved that utilisation of ethanol extract from Sacha inchi leaves is safe but its biological activity, such as its anti-arthritis effect, has not been proven yet. In this study, we evaluated the anti-arthritis effect of ethanol extract from Sacha inchi leaves in Freund’s Complete Adjuvant (CFA)-induced rheumatoid arthritis model and investigated the mechanism of the anti-arthritis effect of the extract.

## MATERIALS AND METHODS

### Specimen Collection and Ethanol Extract Preparation

Fresh Sacha inchi (*P. volubilis*) leaves were provided by the medicinal farm of Inca Sachi Vietnam Joint Stock Company, Pleiku City, Gialai Province, Vietnam in April 2021. Plant material was identified by a taxonomist from the Department of Biotechnology, Institute of Biotechnology and Food Technology, Industrial University of Ho Chi Minh City, Vietnam, and the specimen was deposited in the institutional herbarium with voucher number (No. PV240421VST) for future reference.

**Figure 1:**
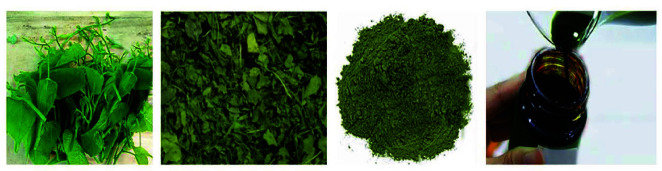
The ethanol extract preparation. Sacha inchi leaves collected from medicinal plant farm in Pleiku city, Gialai province, Vietnam. The leaves were dried until reaching moisture about 10%–12% and ground into powder. The powder was soaked in ethanol, then the extract was obtained through multiple step filtration. The filtrate was subsequently concentrated in a vacuum rotary evaporator.

Leaves were cleaned with tap water, chopped, and dried in an oven at a temperature of 40°C–70°C until reaching moisture about 10%–12%, then ground into powder and stored in a moisture-barrier bag at 25°C to serve for further studies. A total of 1 kg of the leaves powder was soaked with 5 L of absolute ethanol and intermittently shaken in an orbital shaker for 4 days. The solution was filtered through cotton wool and No. 4 Whatman filter paper (Fig. 1). The filtrate was subsequently concentrated in a vacuum rotary evaporator. The crude ethanol extract of Sacha inchi leaves (named EtPV) was collected, weighed, and stored in air- and water-proof packaging, which was kept in the refrigerator at 4°C and wrapped in aluminium foil for preventing light before being used for further experiments.

### Reagents and Chemicals

Complete Freund’s adjuvant (F5881) was purchased from MilliporeSigma, USA; Mobic (meloxicam) was provided from Boehringer Ingelheim, Germany; other chemicals and reagents, such as ethanol, methanol, lead acetate, chloroform, acetic acid, sulfuric acid, gallic acid, quercetin, Folin-Ciocalteu reagent, aluminium chloride, etc. were at analytic grade and obtained from MilliporeSigma (Burlington, MA) unless otherwise stated.

### Phytochemical Screening

To confirm the presence of bioactive compound classes in EtPV, phytochemical screening was performed using standard methods, such as tannins (Braymer’s test), flavonoids (Lead acetate test), saponins (Foam test), steroids (Salkowski test), glycosides (Keller–Kelliani test), alkaloids (Hager’s test), proteins (Xanthoproteic test), carbohydrates (Molisch’s test), phenolic compounds (Ferric chloride test), terpenoids (Liebermann–Burchard test). The details of the phytochemical screening procedure were described in Table 1 (Yadav *et al*. 2010; Sandeep *et al*. 2014; Abdisa & Kenea 2020).

The total polyphenols content in the extract was determined using the Folin-Ciocalteu method. The results were expressed as milligrams of gallic acid equivalent per gram of the dry extract (mg GAE/g). The flavonoid content was measured via the Dowd method (Aryal *et al*. 2019). In concisely, a volume of the extract was added with 0.2 mL of 10% (w/v) aluminum chloride-methanol solution, 0.2 mL of 1 M CH3COOK solution, and distilled water. The absorbance of the mixture or blank at the wavelength 415 nm after being kept in dark at room temperature for 30 min was recorded. The total flavonoid content was determined via a standard curve of quercetin. The results were expressed as milligrams of quercetin equivalent per gram of dry extract (mg QE/g).

### Experimental Animal

Swiss albino mice (male, 30 g–35 g, 7–8 week-old) were obtained from the Institute of Drug Quality Control, Ho Chi Minh City, Vietnam. Mice were housed in the institutional animal care facility of the Industrial University of Ho Chi Minh City for 7 days to acclimatise to the laboratory conditions, six mice per glass cage. The mice had ad libitum access to a standard diet (Anifood, 3.840 kcal/kg, from the Institute of Vaccine and Medical Biologicals, Nha Trang City, Vietnam) and filtered water. During the experiment, all animal received humane care according to the Basel Declaration (2010) and Vietnamese legislation (Law of Animal Husbandry, Law No.32/2018/QH14), and the procedure were in compliance with the Guideline for Preclinical and Clinical Trials of Traditional Medicines and Pharmaceuticals (Decision 141/QD-K2DT of Administration of Science Technology and Training, The Ministry of Health of Vietnam, 2015).

### Dose Selection

The toxicity of ethanol extract of Sacha inchi leaves was investigated in the previous study which confirmed EtPV safety at doses of up to 7,000 mg/kg (acute toxicity test) and 700 mg/kg (sub-chronic toxicity test) (Tran & Tran 2021). Three doses of EtPV (100, 200 and 300 mg/kg), therefore, were selected to be used in this study.

### Evaluation of Anti-Arthritis Activity of EtPV

#### Induction of arthritis

RA was induced by intradermal injection of 0.1 mL of CFA into the footpad of the right hind limbs of the mice. Arthritis was developed in intervals of 7 days. At the end of the 7th day, all the animals developed signs of RA such as swelling, redness, stiffness, and difficulty moving joints. Treatment with EtPV started from the 8th day and continued to the 28th day. The ankle diameter and arthritic score of mice of experimental mice were noted weekly during the experiment.

#### Experimental groups

Experimental groups were designed as described by Cui *et al*. (2019). Briefly, 36 Swiss albino mice were divided into six groups, each group consisting of six mice, as follow: Group 1 (Control group): the healthy mice without CFA induced RA induction were received an equivalent volume of sterile saline; CFA-Untreated group: mice with RA received an equivalent volume of sterile saline; CFA-Mobic group: mice with RA received Mobic (0.2 mg/kg) standard drug CFA-EtPV_100_ group; CFA-EtPV_200_ group; and CFA-EtPV_300_ group: mice with RA received doses 100, 200 and 300 mg EtPV/kg, respectively. All animals were orally administered with indicated doses of vehicle, reference drug, or the extract daily for 3 weeks (from day 8 to day 28).

#### Ankle diameter and arthritic score

The changes in ankle joint diameters of mice were examined weekly by using a Vernier calliper (Mitutoyo, Japan). The arthritic scores were assessed weekly via Cui *et al*. (2019) method. The severity of arthritis in paws was graded from 0 to 4, in which Grade 0 = absence of swelling; Grade 1 = swelling or erythema in one of the toes; Grade 2 = swelling and erythema in one or more toes; Grade 3 = swelling severe and erythema of wrist or ankle; Grade 4 = arthritic swelling in toes or ankle, or gross deformity and inability to use the limb. The arthritic score in each mouse was evaluated according to the variation of erythema, oedema of the injected joint, and the involvement of other non-injected joints (the scores of both hind limbs were counted); therefore, maximum of total score per mouse was 8.

#### Inflammatory parameters

At the end of experiment (day 28), the mice were euthanised using an overdose of carbon dioxide inhalation. The blood samples were collected via cardiac puncture for the determination of hematological, biochemical parameters and inflammatory cytokines assay. The ankle joints were collected for histological analysis and the other parts of the oedematous paws were dissected for antioxidant activity examination. The secondary physical method such as the creation of bilateral pneumothorax was performed to verify the death before carcass disposal.

Half of the blood received from the experimental animal was kept in tubes containing EDTA to test hematological parameters including total erythrocytes (RBC) and leukocytes (WBCs) count, differential leukocytes types count (lymphocytes, monocytes, granulocytes), erythrocyte sedimentation rate-ESR using the Westergren method (Westergren 1957). The other part of the blood was stored in BD Vacutainer™ SST tubes (Thermo, MA, USA), and incubated for 30 min. Subsequently, serum was separated from blood samples by centrifugation (3,000 rpm for 15 min at 4°C). The supernatant was used for the measurement of C-reactive protein (CRP), Rheumatoid factor (RF), and cytokine levels. CRP was determined via Voila *et al*. (1981) procedure and RF was measured with Johnson and Fraulk’s (1976) method. Concentrations of pro-inflammatory cytokines (TNF)-α, interferon (IFN)-γ, interleukin (IL)-1β, IL-6, and anti-inflammatory cytokine IL-10 were determined using a commercial kit according to manufacturer’s instruction (RayBiotech, Inc., Norcross, GA).

### Histopathological Study

Histological analysis was conducted as described by Tran *et al*. (2018) and Liu *et al*. (2017) procedure. In a concise, histological analysis the joints of the right hind limb were separated from the mouse and immersed in formaldehyde (10%) for 24 h. Then the samples were decalcified, washed with running tap water and a series concentration of alcohol and embedded in paraffin. Each specimen was cut into 4–5 μm sections by microtome (Histo-Line, Italy). The tissue was de-waxed, rehydrated, and stained with hematoxylin and eosin. The sections were observed and evaluated under an optical microscope (Nikon, Japan).

### Statistical Analysis

One-way analysis of variance (ANOVA) followed by Multiple Range Test (Stratigraphic Centurion XV software) was used to determine differences among control, CFA-untreated, and CFA-EtPV treated groups. The criterion of statistical significance was set as *p* < 0.05.

## RESULTS AND DISCUSSION

### Phytochemical Screening of EtPV

Even though phytochemical screening of ethanol extract of Sacha inchi leaves has been studied in a previous study, different time points and locations of the specimen collection as well as the difference in extraction techniques could alter the chemical composition of the extract (Tran & Tran 2021). Hence, the phytochemical screening to standardise extract before further experiment is an indispensable task. The results of preliminary analysis of the phytochemical composition of EtPV showed the presence of alkaloids, flavonoids, tannins, phenolic compounds, terpenoids, saponins, steroids, glycosides, carbohydrates in the extract, whereas protein was absent in the extract. The content of polyphenols in EtPV was 42.67 ± 0.81 mg GAE/g, while total flavonoid content was 8.85 ± 0.08 mg QE/g. These results are in line with the previous reports (Nascimento *et al*. 2013; Castillo Saavedra *et al*. 2010). Nascimento *et al*. (2013) observed the presence of colour bands characterised by phenolic compounds, terpenoids, terpenes, steroids, carbohydrates and flavonoids in the ethanol extract of *P. volubilis*. Besides, Castillo Saavedra *et al*. (2010) indicated saponins, flavonoids, steroids, alkaloids and tannins as constituents of the extract and amino acid was not detectable. Many studies have suggested that both flavonoids and polyphenols could regulate inflammatory response by targeting several mechanisms and signal pathways (dos Santos *et al*. 2006; Ambriz-Pérez *et al*. 2016; Ferraz *et al*. 2020). According to Ambriz-Pérez *et al*. (2016), phenolic compounds not only inhibit the production or activity of some inflammatory mediators such as cyclooxygenase-derived prostaglandin E2 and pro-inflammatory cytokines (IL6, TNFα, etc.) but also alter of the synthesis process of eicosanoids, activation of immune cells, and expression of nitric oxide synthase and cyclooxygenase-2. Moreover, nuclear factor-kB or Nrf-2, two well-known transcriptional factors associated with inflammatory and antioxidant pathways, could be regulated by phenolic compounds (Ambriz-Pérez *et al*. 2016). Recently, Al-Khayri *et al*. (2022) have proposed that flavonoids could reduce inflammatory response via many mechanisms, for instance, inhibition of MAPK and NF-kB pathways or activator protein-1 transcription factor. Quercetin, a well-known member of flavonoids, has been documented for its suppressive effect on neutrophil recruitment and actin polymerization (Souto *et al*. 2011; Ferraz *et al*. 2020; Al-Khayri *et al*. 2022). In addition, apigenin, another member of flavonoids, could inhibit p65 phosphorylation (the subunit of the NF-kB inflammatory pathway) and suppress nitrite oxide and cyclooxygenase-2 production in macrophages (Ginwala *et al*. 2019). Many flavonoids, including luteolin, genistein and fisetin, have also been reported for their suppression of inflammatory cytokines, such as TNFα and IL6 production (Al-Khayri *et al*. 2022). The presence of bioactive compounds, such as flavonoids and phenolic compounds, which could regulate the inflammatory response, implies the basis for the anti-arthritis effect of the extract.

### The Effect of EtPV Extract on Ankle Diameter and Arthritic Score

Ankle diameter and arthritic score are considered useful indices to evaluate the severity of RA. The increase in ankle diameter has been recorded from day 0 to day 7 and reached the maximum on day 28 in RA mice (5.59 ± 0.36 mm) and was significantly higher than those of the control group (0.65 ± 0.19 mm, *p* ˂ 0.05). Additionally, the arthritic score was found to be maximum (7.33 ± 0.82) on the 28th day in RA mice and in contrast to healthy mice. The results are in agreement with the macroscopic investigation of the hind paws of the experimental mice, in which paws of RA mice were characterised by redness, swelling, and oedema at joints (Fig. 3). The data from this study was in line with the results of Zhao *et al*. (2016) study, in which the paw swelling reached the peak at 14 days after CFA injection, and the arthritis score of the RA model peaked around day 18. Injection of CFA results in the release of histamine, prostaglandins, and kinins into the bloodstream, which in turn enhances the permeability of blood vessels and blood flow into inflamed areas and leads to oedema (Triastuti *et al*. 2021).

**Figure 2:**
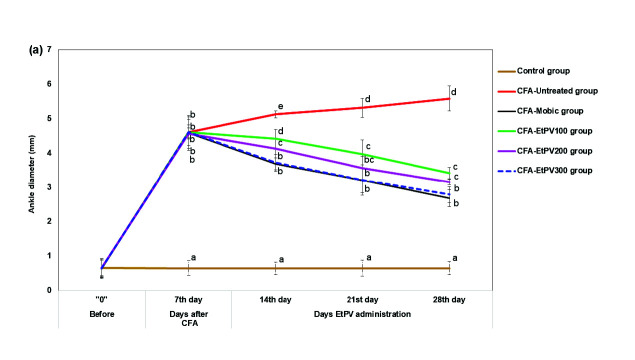

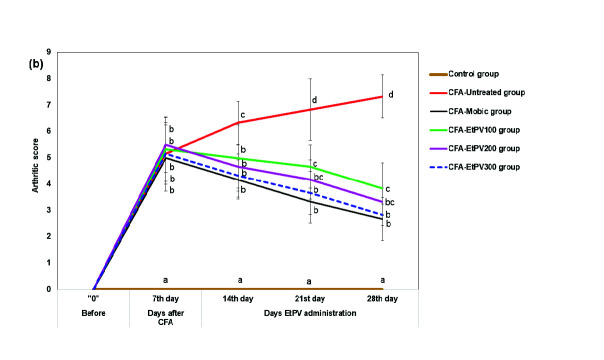
The therapeutic effect of EtPV on ankle diameters, arthritis score in mice with CFA-induced arthritis. (A) Ankle diameters, (B) Arthritis scores. Values were expressed as Mean ± SD (*n* = 6) and the letters a, b, c and d denote the significant difference among groups.

**Figure 3:**
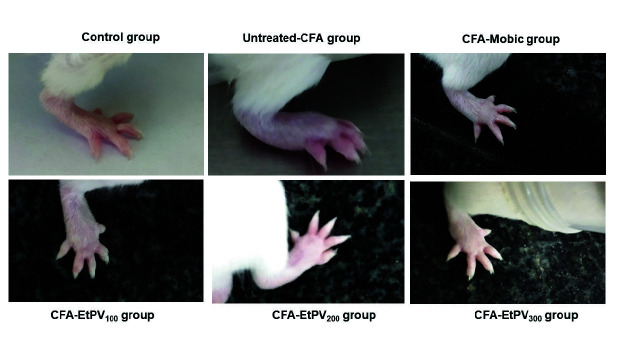
Macroscopic examination of experimental mice. Representative pictures of paws mice from each group (control, CFA-induced RA model, CFA-Mobic, CFA-EtPV_100_/EtPV_200_/EtPV_300_) were presented. Briefly, the paw of RA mice was redness, swelling, and oedematous, whereas treatment with Mobic/EtPV_100_/EtPV_200_/EtPV_300_ showed significant differences and alleviation of paw swelling.

On the other hand, all the treatments with various doses of EtPV reduce the paw swelling (ankle diameter) as well the severity of arthritis (arthritis score) in a dose-dependent and time-dependent manner (Figs. 2A, 2B). For instance, treatment with Mobic showed a remarkable decline in both ankle diameter (2.69 ± 0.25 mm) and arthritis score on the 28th day (2.67 ± 0.82) in comparison with those of CFA induced model (5.59 ± 0.36 mm and 7.33 ± 0.82, respectively, *p* < 0.05), but they were still higher those of control group (*p* < 0.05). On the contrary, treatment with EtPV at the doses of 200 and 300 mg/kg B.W. could reverse swelling and redness of paw induced by CFA which showed via the reduction of arthritis scores (3.33 ± 0.52 and 2.83 ± 0.41, respectively) and morphological changes and their efficacy was similar with Mobic treated group (Figs. 2B and 3). In addition, CFA-EtPV_100_ group also showed a remarkable decline of arthritis scores (3.83 ± 0.98) as compared to CFA-group (7.33 ± 0.82) but its efficacy was lower that of Mobic treated group (2.67 ± 0.82, *p* < 0.05). Among the three groups of treatment after 28 days, only the ankle diameters of mice in the CFA-EtPV300 group were comparable with those of the CFA-Mobic group (2.69 ± 0.25 versus 2.79 ± 0.23 mm, *p* > 0.05), whereas those of other groups, including CFA-EtPV_100_ and CFA-EtPV_200_ groups were toward the normal range but still different with reference drug-treated group (Fig. 2A). That suggests the dose of the extract of 300 mg/kg B.W. as the optimal dose among three doses of treatment in term of alleviation of oedema in the paws.

### The Effect of EtPV Extract on Hematological and Biochemical Parameters

The hematological and biochemical parameters are important indicators to evaluate the effect of the extract on the treatment for RA. The RBC count was drastically reduced (about 57%), and at the same time increased the number of leukocytes in the RA group (1.83 times) versus the control group (*p* < 0.05). Anaemia is a common complication of rheumatoid arthritis syndrome, due to the correlation of elevation IL6 and the production of hepcidin, an iron regulatory hormone, which prevents iron absorption in the duodenum (Ruckmani *et al*. 2017). On the other hand, EtPV recovered anaemia induced by RA and reduced considerably the number of leukocytes as compared to those of the RA model in a dose-dependent manner (*p* < 0.05) (Table 2). The high dose of the extract (300 mg/kg B.W.) could improve the RBC count almost similar to Mobic treat mice (*p* > 0.05) whereas RBC counts of other lower doses of EtPV treatment (100 and 200 mg/kg B.W.) were lower than of Mobic treat group (*p* < 0.05). Among three doses of treatment, the number of leukocytes of the CFA-EtPV_300_ group was significantly lower than the other doses (100 and 200 mg/kg B.W.) and tended toward the range of reference drug-treated mice. These data are in line with the findings of previous studies. For instance, Zhang *et al*. (2020) indicated that CFA decreased the number of erythrocytes (about 67%) and elevated the number of leukocytes (about two times) as compared to control mice, while treatment with ginkgolide acid, a natural phenolic antioxidant, could increase the RBC count and reduce the WBC count of CFA treated mice in a dose-dependent manner. Recently, Jan *et al*. (2022) also observed a similar phenomenon in terms of the RBC and WBC counts when they investigated the anti-arthritis effect of aqueous and ethanol extract of Euphorbia helioscopia in CFA-induced mice.

In addition, ESR, RF and CRP have manifested chronic inflammation conditions and the severity of arthritis. After 28 days of CFA administration, CFA mice showed an increase of ESR and CRP and RF levels of the disease control group were significantly elevated versus the control group (*p* < 0.05). In a previous study, Ananth *et al*. (2016) observed an elevation of CRP, RF levels, and ESR after 35 days of CFA treatment. This indicated arthritis model in the present study was successfully established. Treatment with EtPV not only improved hematological parameters but also showed a significant reduction of some arthritis indicators such as levels of CRP, RF and ESR (Table 2), which was comparable with the results of previous reports (Ananth *et al*. 2016; Tang *et al*. 2021). For example, treatment with *Pergularia daemia* extract results in an increase in RBC count and a decline of WBC count and other biochemical parameter including RF, CRP levels, and ESR, which implies the protective effect of *P. daemia* extract against CFA-induced arthritis (Ananth *et al*. 2016). Furthermore, 3,5,7,3′,4′-Pentahydroxy flavone, a flavonoid isolated from *Madhuca indica*, also could reduce the increase of CRP, RF levels and ESR induced by CFA treatment (Tang *et al*. 2021). Of note, only the dose of 300 mg extract/kg body weight exhibited a similar efficacy with reference drug (Mobic, 0.2 mg/kg body weight) in terms of reduction of lymphocytes, monocytes, granulocytes, ESR, CRP and RF (*p* > 0.05). Besides that, lower doses of the extract (100 and 200 mg extract/kg body weight) exhibited a lower number of lymphocytes, monocytes, granulocytes, ESR, CRP, and RF as compared to RA-group (*p* < 0.05) but still higher than those of Mobic treated mice (*p* < 0.05). That indicates the dose of 300 mg EtPV/kg B.W. is the optimal dose among the three doses of treatment.

### Histopathological Analysis

**Figure 4:**
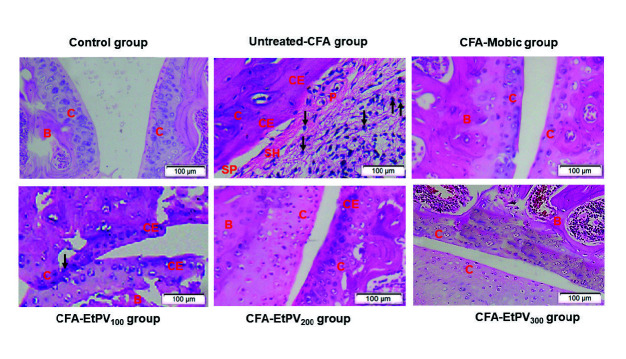
The therapeutic effect of EtPV and Mobic on histopathological features of joints (stained with hematoxylin and eosin); Symbols: B = Bone; C = Cartilage; CE = Cartilage erosion; SP = Synovial space; SH = Synovial hyperplasia; P = Pannus; → cellular infiltration. Adjuvant-induced arthritis group is characterised by a distinct bone and cartilage erosion, synovial hyperplasia and pannus formation, destruction of joint structure with immune cells infiltration, whereas Mobic or EtPV treatment reduces the immune cells infiltration and improves the joint structure.

The results from the histological examination also support the biochemical and hematological analysis. Histological analysis of ankles of healthy mice revealed no cartilage destruction and no signs of inflammation or there was no distortion in the structure of joints. Joints of RA mice noticed distinct bone and cartilage erosion, synovial hyperplasia and pannus formation, destruction of joint space with immune cell infiltration. In Mobic-treated mice, joint sections exhibited an increase in the smooth articular surface and articular cartilage layer, normal joint space, and reduction in synovial hyperplasia and pannus formation (Fig. 4). The therapeutic administration with EtPV 300 mg/kg B.W. (EtPV_300_) had remarkably changed the histological in the experimental mice with the reduction in synovial hyperplasia and pannus formation, normal structure in space of joint, no longer presence of inflammatory cells in articular cartilage, which are consistent with the results from hematological and biochemical parameters. On the other hand, treatment with lower doses of EtPV including EtPV_100_ and EtPV_200_ showed a rough surface of cartilage, which indicated cartilage erosion. These data also prove the dose of 300 mg/kg B.W. as the optimal dose among three doses of treatment. To elucidate the anti-arthritis effects of extract, we eventually investigated the production of some pro and anti-inflammatory cytokines, one of the key factors contributing to RA pathogenesis.

### The Effect of EtPV Extract on Inflammatory Cytokines

**Figure 5:**
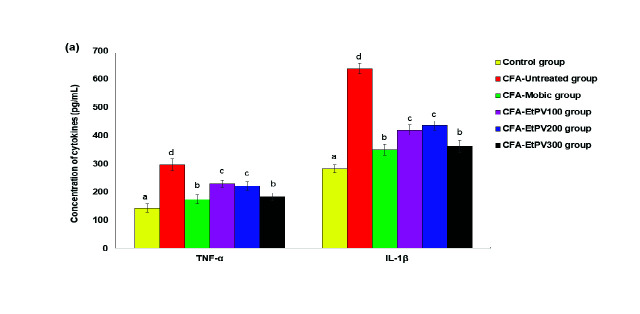

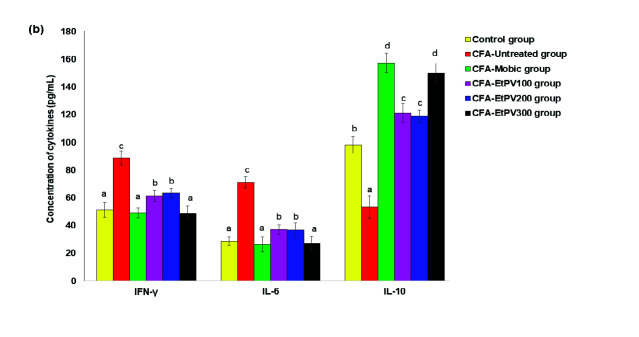
The therapeutic effect of EtPV on concentration of: (A) TNF-α and IL-1β, and (B) IFN-γ, IL-6 and IL-10 in mice with CFA-induced arthritis. Values were expressed as Mean ± SD (*n* = 6) and the letters a, b, c, and d denote the significant difference among groups.

Numerous inflammatory cytokines such as TNF-α, IL-1β, and IL-6, etc. may participate in the damaging inflammation in RA. The remarkable elevation of pro-inflammatory cytokines was perceived in the RA mice in contrast to the control mice (*p* < 0.001). The concentrations of pro-inflammatory cytokines TNF-α, IL-1β, IFN-γ, and IL-6 were elevated in the serum of mice RA. Meanwhile, the concentration of IL-10, an anti-inflammatory cytokine, was decreased (Fig. 5). The experimental groups administered EtPV caused a significant reduction of TNF-α, IL-1β, IFN-γ, IL-6 and significantly restored IL-10 levels as compared to RA model (*p* < 0.05). For example, TNF-α, IL-6, and IL-1β levels in the serum of the mice in the CFA-EtPV_300_ group were 182.84 ± 14.50 pg/mL, 27.47 ± 4.63 pg/mL, and 362.58 ± 21.57 pg/mL significant decrease versus levels of TNF-α, IL-6, and IL-1β in the disease group (296.27 ± 21.37 pg/mL, 71.35± 4.17 pg/mL and 636.57 ± 18.23 pg/mL, *p* < 0.05, respectively). In addition, IFN-γ of the CFA-EtPV_300_ group (48.89 ± 5.19 pg/mL) was significantly decreased as compared to the disease group (88.79 ± 5.11 pg/mL, *p* < 0.05) and had a tendency toward the normal range (51.33 ± 5.44 pg/mL, *p* > 0.05). At the same time, IL-10 levels of the CFA-EtPV_300_ group were significant increase (149.89 ± 7.34 pg/mL) versus levels of IL-10 in the RA group were 53.42 ± 8.18 pg/mL (*p* < 0.05). Of note, TNF-α, IL-1β, IFN-γ, IL-6 and IL-10 levels of the CFA-EtPV_300_ group were comparable with those of CFA-Mobic treated mice (Fig. 5, *p* > 0.05). On the contrary, inflammatory cytokines (TNF-α, IL-1β, IFN-γ and IL-6 levels) in lower doses of the extract-treated mice (EtPV_100_ and EtPV_200_ groups) were higher than those of Mobic and EtPV_300_ treated groups. Besides that, anti-inflammatory cytokine (IL-10 level) in lower doses of the extract-treated mice (EtPV_100_ or EtPV_200_ groups) was lower than that of the Mobic-treated group. That implies the efficacy of EtPV_300_ is equivalent to Mobic (0.2 mg/kg body weight), whereas the efficacies of EtPV_100_ and EtPV_200_ are lower than the Mobic (0.2 mg/kg body weight) treatment.

RA initiates by the T cell-mediated immune response that stimulates the release of pro-inflammatory cytokines such as TNF-α, IL-1β, IFN-γ and IL-6. These cytokines were promoting antibody formation, which aggravates inflammation, bone erosion, and cartilage destruction in joint tissues. Some inflammatory cytokines, for example, IL-1β and IL-6, could stimulate NF-κB (transcriptional factor) resulting in the activation of osteoclasts and enhancing ROS production which leads to promoting bone resorption (Wojdasiewicz *et al*. 2014). NF-κB also increased the symptoms of rheumatoid arthritis by supporting the Th1 response. Previous reports have demonstrated that IL-10 could suppress the pro-inflammatory cytokines TNF-α, IL-6, IFN-γ and IL-1β as well as inhibit the synthesis of nitric oxide, gelatinase, and collagenase (Chernoff *et al*. 1995). In this study, EtPV treatment not only reduces the elevation of pro-inflammatory cytokines such as TNF-α, IL-1β, IFN-γ and IL-6 but also enhances the anti-inflammatory cytokine, IL-10. The data suggests that the extract exhibits an immunomodulatory effect on inflammatory cytokines, which underlies its beneficial effect and improvement on the arthritis model. For instance, earlier studies have documented that CRP levels in serum are closely related to the RA disease progression rate and CRP is released from the liver in response to IL-6 activity during inflammation; thus, it is no surprise that the extract treatment reduces both IL-6 and CRP levels (Pope & Choy 2021). Furthermore, IL-6 also can regulate anaemia through hepcidin, an iron-regulatory hormone, which blocks the release of iron from macrophages and iron absorption at the duodenum (Ruckmani *et al*. 2017). The extract could increase the IL-6 level; consequently, it improves anaemia and erythrocyte number as well. The results from this study are consistent with Ambulay *et al*. (2020) research, in which the author indicated that Sacha inchi seed oil (*P. huayllabambana*) could modulate the oxidative stress and inflammatory cytokines (TNF-α, IL-6, IL-10 and IL-4) production. The majority of studies on Sacha inchi about its bioactivities and beneficial effect have been performed on seed oil but not the Sacha inchi leaves. Hence, these findings proved for anti-inflammatory and anti-arthritis effects of Sacha inchi leaves for the first time. Moreover, the data from this study also corroborate the growing evidence for using plant extracts or derivatives as anti-inflammatory and anti-arthritis medicines. In the previous study, Allam *et al*. (2016) proposed that ellagic acid, a natural phenolic compound, could modulate pro-inflammatory cytokines (TNF-α, IL-1β and IL-17) and enhance anti-inflammatory cytokines, such as IL-10, which in turn could ameliorate CFA induced arthritis in mice. Additionally, mandarin peel extract also has the ability to suppress ankle diameter, serum pro-inflammatory cytokines (TNF-α and IL-1β) as well as enhanced anti-cytokines (IL-10, IL-4) (Sakr *et al*. 2021). Some flavonoids also could modulate signalling pathways such as NF-κB pathways, which leads to suppression of inflammatory response. For instance, Lee *et al*. (2012) revealed that sulfuration, a flavonoid extracted from *Rhus verniciflua*, alleviated inflammatory response, pro-inflammatory cytokines production, and joint destruction in collagen-induced arthritis mice via its inhibitory effect on NF-κB pathway. Furthermore, rhoifolin, a natural flavonoid found in *Rhus succedanea*, also suppresses the NF-κB pathway, which results in the modulation of oxidative stress and pro-inflammatory cytokines production (TNF-α, IL-6 and IL-1β) (Peng *et al*. 2020).

## CONCLUSION

The data from this study suggest ethanol extract from Sacha inchi leaves is a promising remedy for rheumatoid arthritis, which exhibits anti-arthritis activity with an optimal dose of about 300 mg/kg. This study also reveals the presence of some bioactive compounds in the extract including saponins, alkaloids, flavonoids, tannins, terpenoids and phenolic compounds, which in turn exhibit anti-arthritis effect through modulation of inflammatory cytokines. These findings not only provide the scientific evidence for the utilisation of Sacha inchi leaves, an inexpensive and abundant material of *P. volubilis*, to isolate bioactive compounds for anti-inflammatory effect but also prove the effectiveness of ethanol extract *P. volubilis* on rheumatoid arthritis treatment, although further experiments and clinical studies need to be employed.

## Figures and Tables

**Table 1:** Phytochemical screening procedure of the extract.

Phytochemical test	Procedure	Interference for presence of phytochemicals	Reference
Tannins (Braymer’s test)	The extract (1 mL) + water (2 mL) + a few drops of ferric chloride solution (5%).	Formation of green precipitate.	Abdisa & Kenea (2020)
Flavonoids (Lead acetate test)	The extract (1 mL) + a few drops of lead acetate solution (10%).	Formation of yellow precipitate.	Abdisa & Kenea (2020)
Saponins (Foam test)	The extract (1 mL) + water (20 mL), the mixture was shaken in measuring cylinder for minutes.	Appearance of stable foam.	Abdisa & Kenea (2020)
Steroids (Salkowski test)	The extract was mixed with chloroform, added a few drops of concentrated sulfuric acid.	Appearance of a red colouration in lower layer.	Yadav *et al*. (2010)
Glycosides (Keller-Killiani test)	The extract (5 mL) + glacial acetic acid (2 mL) + a few drops of FeCl3 + concentrated sulfuric acid.	Appearance of a brown ring in the interface.	Abdisa & Kenea (2020)
Carbohydrates (Molisch’s test)	The extract (2 mL) + a few drops of Molisch’s solution + concentrated sulfuric acid.	Appearance of a purple coloration of the interface.	Abdisa & Kenea (2020)
Phenolic compounds (Ferric chloride test)	The extract (0.5 mL) + 5 mL distilled water + a few drops of 5% ferric chloride solution.	Appearance of a dark green colouration.	Abdisa & Kenea (2020)
Alkaloids (Hager’s test)	The extract (2 mL) + a few drops of Hager’s reagent.	Formation of yellow precipitate.	Sandeep *et al*. (2014)
Proteins (Xanthoproteic test)	The extract (3 mL) + 1 mL concentrated H_2_SO_4_. The mixture was boiled and added 1 mL NH_4_OH solution.	The white precipitate tuned to yellow after boiling. Appearance of orange colouration after adding NH_4_OH.	Sandeep *et al*. (2014)
Terpenoids (Liebermann–Burchard test)	The extract (1 mL) was mixed with chloroform + a few drops of acetic anhydride. The mixture was boiled and rapidly cooled + concentrated H_2_SO_4_.	Appearance of a deep red colouration.	Yadav *et al*. (2010)

**Table 2:** The therapeutic effect of EtPV on hematological and biochemical parameters of RA mice.

Parameters	Control group	CFA-Untreated group	CFA-Mobic group	CFA-EtPV_100_ group	CFA-EtPV_200_ group	CFA-EtPV_300_ group
RBC (×10^6^cells/mm^3^)	8.46 ± 0.32^e^	4.81 ± 0.62^a^	7.75 ± 0.61^d^	6.98 ± 0.43^bc^	6.52 ± 0.57^b^	7.36 ± 0.59 ^cd^
ESR (mm/hr)	4.42 ± 0.22^a^	7.32 ± 0.51^d^	4.59 ± 0.34^ab^	5.89 ± 0.43^c^	6.02 ± 0.49^c^	4.94 ± 0.38^b^
WBC (×10^3^ cells/mm^3^)	5.36 ± 0.39 ^a^	9.78 ± 0.42^e^	6.34 ± 0.24^b^	7.81 ± 0.18^d^	7.75 ± 0.16^d^	6.76 ± 0.21^c^
Lymphocytes (×10^3^ cells/mm^3^)	3.26 ± 0.49^a^	5.96 ± 0.22^d^	3.87 ± 0.11^b^	4.77 ± 0.14^c^	4.73 ± 0.15^c^	4.13 ± 0.18^b^
Monocytes (×10^3^ cells/mm^3^)	1.33 ± 0.23^a^	2.44 ± 0.14^d^	1.58 ± 0.07^b^	1.95 ± 0.08^c^	1.93 ± 0.07^c^	1.69 ± 0.06^b^
Granulocytes (×10^3^ cells/mm^3^)	0.71 ± 0.11^a^	1.38 ± 0.07^d^	0.89 ± 0.06^b^	1.09 ± 0.08^c^	1.09 ± 0.07^c^	0.94 ± 0.07^b^
CRP (mg/L)	0.14 ± 0.04^a^	8.21 ± 1.25^d^	2.04 ± 0.36^b^	3.47 ± 0.41^c^	3.45 ± 0.52^c^	2.14 ± 0.39^b^
RF (mg/L)	0.16 ± 0.05^a^	9.34 ± 1.36^d^	2.46 ± 0.38^b^	3.75 ± 0.43^c^	3.69 ± 0.55^c^	2.64 ± 0.34^b^

*Notes*: Values were expressed as Mean ± SD (*n* = 6) and the letters a, b, c, d, e in a row denote the significant difference among groups.
